# Targeted long-read sequencing identifies a novel 1.3 kb *FGG* deletion associated with variable bleeding and thrombotic phenotypes in hypofibrinogenemia

**DOI:** 10.1016/j.rpth.2026.107001

**Published:** 2026-10-01

**Authors:** Alexander Couzens, Frédéric Masclaux, Baptiste Micheli, Michel Guipponi, Alexandre Butelet, Colette Ballan-Legin, Alessandro Casini, Marguerite Neerman-Arbez

**Affiliations:** 1 Department of Genetic Medicine and Development, Faculty of Medicine, University of Geneva, Geneva, Switzerland; 2 Medical Genetics Department, University Hospitals of Geneva, Geneva, Switzerland; 3 Centre for Rare Bleeding Disorders, Reims University Hospital, Reims, France; 4 Division of Angiology and Haemostasis, University Hospitals of Geneva, Geneva, Switzerland

**Keywords:** fibrinogen, fibrinogen deficiency, genomic structural variation, nanopore sequencing, phenotypic variability

## Abstract

**Background:**

Congenital fibrinogen disorders are rare inherited disorders of fibrinogen quantity or function and are associated with variable bleeding and thrombotic phenotypes. They are caused by variants in *FGA*, *FGB*, or *FGG*. Most reported pathogenic variants are single-nucleotide variants or small insertions/deletions, whereas structural variants likely remain under-reported because short-read sequencing often cannot detect or fully resolve them.

**Objectives:**

We aimed to identify and characterize the genetic cause of hypofibrinogenemia in three probands and their families and to investigate additional genetic variation potentially contributing to clinical variability.

**Methods:**

Whole-exome sequencing (WES) identified variants in fibrinogen and other coagulation and fibrinolysis genes. Targeted Oxford Nanopore long-read sequencing resolved a suspected structural variant, defined its breakpoints, and enabled haplotype phasing across families. Kinship analysis assessed relatedness between probands.

**Results:**

WES showed reduced read depth across *FGG* in three apparently unrelated probands. Long-read sequencing resolved a novel 1.3 kb deletion spanning the 5′ end of *FGG*, with breakpoints in A/T-rich low-complexity sequences. All three probands shared an identical 18 kb haplotype carrying the deletion, supporting inheritance from a common founder. Phenotypes ranged from mild bleeding to recurrent thrombosis. Compound heterozygosity for the deletion and *FGG* p.Ala108Gly was associated with a more marked reduction in fibrinogen levels than heterozygosity for the deletion alone. Additional rare variants in hemostasis-related genes were identified, although their contribution to phenotypic variability remains uncertain.

**Conclusion:**

Long-read sequencing enables precise characterization and phasing of structural variants in congenital fibrinogen disorders, whereas WES facilitates investigation of additional genetic variation that may contribute to disease expression.

## Essentials

• Few structural variants have been identified in inherited fibrinogen disorders.
• We used long-read DNA sequencing to study three families with low fibrinogen levels.
• A 1.3 kb deletion in one of the fibrinogen-encoding genes was found in affected individuals.
• Shared DNA around the deletion suggested a common ancestor, but symptoms varied between families.

## 1 Introduction

Fibrinogen is an abundant plasma protein that plays a central role in hemostasis as the soluble precursor of fibrin, which polymerizes to form the scaffold that stabilizes a blood clot. It is synthesized in hepatocytes as a hexamer composed of 2 sets of Aα, Bβ, and γ chains, encoded by the *FGA*, *FGB*, and *FGG* genes on chromosome 4. In healthy individuals, plasma concentrations are tightly regulated within a typical range of 2.0 to 4.0 g/L [1], [2]. Alterations in fibrinogen levels, function, or localization can have important consequences for hemostasis and human health [2], [3]. Congenital fibrinogen disorders (CFDs) are a heterogeneous group of diseases classified as either quantitative or qualitative according to antigenic and functional fibrinogen levels [4], [5]. Quantitative CFDs include afibrinogenemia, characterized by undetectable circulating fibrinogen, and hypofibrinogenemia, defined by reduced fibrinogen levels and further subclassified as severe (<0.5 g/L), moderate (0.5-0.9 g/L), or mild (1.0 g/L to the lower limit of normal) [4]. In contrast, qualitative CFDs include dysfibrinogenemia, characterized by normal antigenic fibrinogen levels with impaired functional activity, and hypodysfibrinogenemia, which combines quantitative deficiency with qualitative dysfunction. Consistent with the essential role of fibrinogen in clot formation and stability, the principal manifestations of CFDs are bleeding and, paradoxically, thrombosis [5], [6], [7].

CFDs are caused by pathogenic variants in *FGA*, *FGB*, or *FGG*. Most reported CFD-associated variants are single-nucleotide variants or small insertions/deletions [8], [9]. In contrast, structural variants (≥50 bp), although a major source of genetic variation, appear comparatively uncommon in CFDs, with only a limited number described to date [10], [11], [12], [13], [14], [15], [16]. This apparent rarity may partly reflect the historical reliance on Sanger sequencing for molecular investigation of CFDs and the limitations of short-read next-generation sequencing approaches, including targeted gene panels and whole-exome sequencing (WES). These widely used approaches have reduced sensitivity for detecting complex structural variants and limited ability to resolve breakpoints, particularly in repetitive, intronic, or intergenic regions that are prone to rearrangement [15], [16].

In contrast, long-read sequencing technologies, including Oxford Nanopore Technologies (ONT) and PacBio, enable improved detection and breakpoint resolution of complex structural variants, helping to identify sequence features that may contribute to rearrangement formation [17], [18], [19]. By spanning multiple variants within individual reads, long reads also enable haplotype phasing across large genomic distances, allowing variants to be assigned to the same or opposite chromosomes without requiring parental information. This is useful for distinguishing variants acting in cis from those acting in trans, which can be important for genotype-phenotype interpretation, particularly in compound heterozygous cases. Despite its increasing accessibility, long-read sequencing has not yet been used to identify, fully characterize, and phase disease-associated variants in CFDs.

Although WES is limited for resolving complex structural variation, it remains well suited to the parallel identification of rare and common coding variants in hemostasis-related genes that may modify clinical expression. This is particularly relevant in CFDs, where individuals carrying the same disease-causing variant may exhibit substantial differences in clinical presentation, including within the same family [20], [21]. Such variability suggests that additional genetic, as well as environmental, modifiers may further alter the hemostatic balance in affected individuals. For example, the well-established prothrombotic variants in *F5* Leiden and *F2* G20210A are known to increase thrombotic risk in the general population and may therefore contribute to phenotypic variability among patients with CFDs [22].

Here, we report a novel *FGG* deletion fully resolved by long-read sequencing in 3 apparently unrelated families with hypofibrinogenemia. Haplotype analysis demonstrated that the deletion was carried on a common haplotype, supporting a founder effect. Despite sharing the same primary fibrinogen defect, affected individuals exhibited differences in fibrinogen levels and clinical presentation, prompting investigation of additional genetic variation identified by WES. These findings underscore the complementary value of long-read sequencing for characterizing structural variants and haplotypes in CFDs, and of WES for identifying additional candidate variants of potential clinical relevance.

## 2 Methods

### 2.1 Patient samples

This study was performed with institutional review board approval and with written informed consent from all patients, in accordance with the Declaration of Helsinki.

### 2.2 Whole-exome sequencing

WES was performed using the Twist Comprehensive Exome capture kit (Twist Bioscience) followed by sequencing on an Illumina NovaSeq platform. Read alignment and variant calling were carried out using bwa-mem v0.7.18, sambamba v1.0.1, and DeepVariant v1.5.0 (WES model). Variants were annotated using ANNOtate VARiation (ANNOVAR; v07.06.2020) with the UCSC RefSeq database (refGene; downloaded 12.08.2025). Variants were queried against dbSNP build 156, gnomAD v2.1.1, ClinVar (2025), and the Human Gene Mutation Database (HGMD; 2016). In silico pathogenicity predictions for missense variants were obtained using Sorting Intolerant From Tolerant (SIFT), PolyPhen-2, MutationTaster, Combined Annotation Dependent Depletion (CADD), AlphaMissense, and Rare Exome Variant Ensemble Learner (REVEL). Copy number variant (CNV) analysis was performed using an in-house read-depth–based algorithm, as previously described by Van Heurck et al. [23], which has been successfully applied to identify large deletions in hypofibrinogenemia [15].

### 2.3 Oxford Nanopore Technologies long-read sequencing

Long-range polymerase chain reaction (PCR) amplicons (∼20 kb) covering the fibrinogen genes were generated from genomic DNA using KOD One PCR Master Mix (Toyobo). Libraries were prepared using the Rapid Sequencing Kit v14 (SQK-RAD114) and sequenced on a MinION Mk1C device with Flongle flow cells (R10.4.1), all from Oxford Nanopore Technologies. Basecalling was performed using EPI2ME Labs wf-basecalling v1.3.0, and reads were aligned to GRCh38/hg38 (GCA_000001405.15). Variant calling was performed using EPI2ME Labs wf-human-variation v2.2.5. Additional calling was performed using DeepVariant v1.6.0, and variants were annotated using Ensembl VEP v105.0. Phasing was carried out using WhatsHap v2.3, and Binary alignment map (BAM) and variant call format (VCF) files were visualised in Integrative Genomics Viewer (IGV) v2.17.4. To assess whether the deletion had previously been reported, its genomic coordinates were queried for matching or overlapping structural variants in the Database of Genomic Variants, the Database of Genomic Variation and Phenotype in Humans Using Ensemble Resources (DECIPHER), gnomAD v4.1.1, the gnomAD SV/CNV v4.1.0 dataset, and NCBI dbVar (accessed July 24, 2026).

### 2.4 Sanger sequencing

PCR and Sanger sequencing were performed in individuals who were not included in the WES or ONT sequencing analyses. Primers were designed to span the *FGG* deletion breakpoints, enabling discrimination between the deleted and wild-type alleles. PCR products were analyzed by agarose gel electrophoresis, yielding amplicons of approximately 1.2 kb for the deletion allele and 2.5 kb for the wild-type allele. PCR products were subsequently purified and sequenced to confirm the presence or absence of the deletion and provide further verification of the breakpoint sequences.

### 2.5 Variant interpretation

Variants of interest were interpreted using population frequency data, published literature, computational predictions, and available segregation data. Relevant American College of Medical Genetics and Genomics (ACMG) and Association for Molecular Pathology (AMP) guidance was applied where appropriate.

### 2.6 Kinship analysis

Genetic relatedness between probands was assessed using the pairwise kinship coefficients algorithm calculated with the KING toolset (–kinship option), as described in the software manual (https://www.kingrelatedness.com/). A merged variant file including the study participants and >1000 unrelated reference individuals was used to avoid population structure effects and to minimize the impact of population stratification.

### 2.7 Protein modeling

The thrombomodulin protein structure was modeled using AlphaFold3 due to the absence of an available X-ray crystal structure for the region of interest. The predicted structure was visualized in PyMOL (v3.1.6.1), and in silico mutagenesis was performed to substitute glutamic acid with lysine at position 150 of the mature protein, corresponding to p.Glu168Lys in the full-length sequence (accounting for the 18–amino-acid signal peptide). The wild-type and variant structures were subsequently superimposed and displayed side by side to enable structural comparison.

## 3 Results

Probands F1-II-6, F2-II-11, and F3-II-3, from 3 apparently unrelated families (family 1 [F1], family 2 [F2], and family 3 [F3]), were referred for genetic investigation because of reduced fibrinogen levels consistent with hypofibrinogenemia. Familial screening was undertaken where possible (Figure 1 and Table).

**Figure 1:**
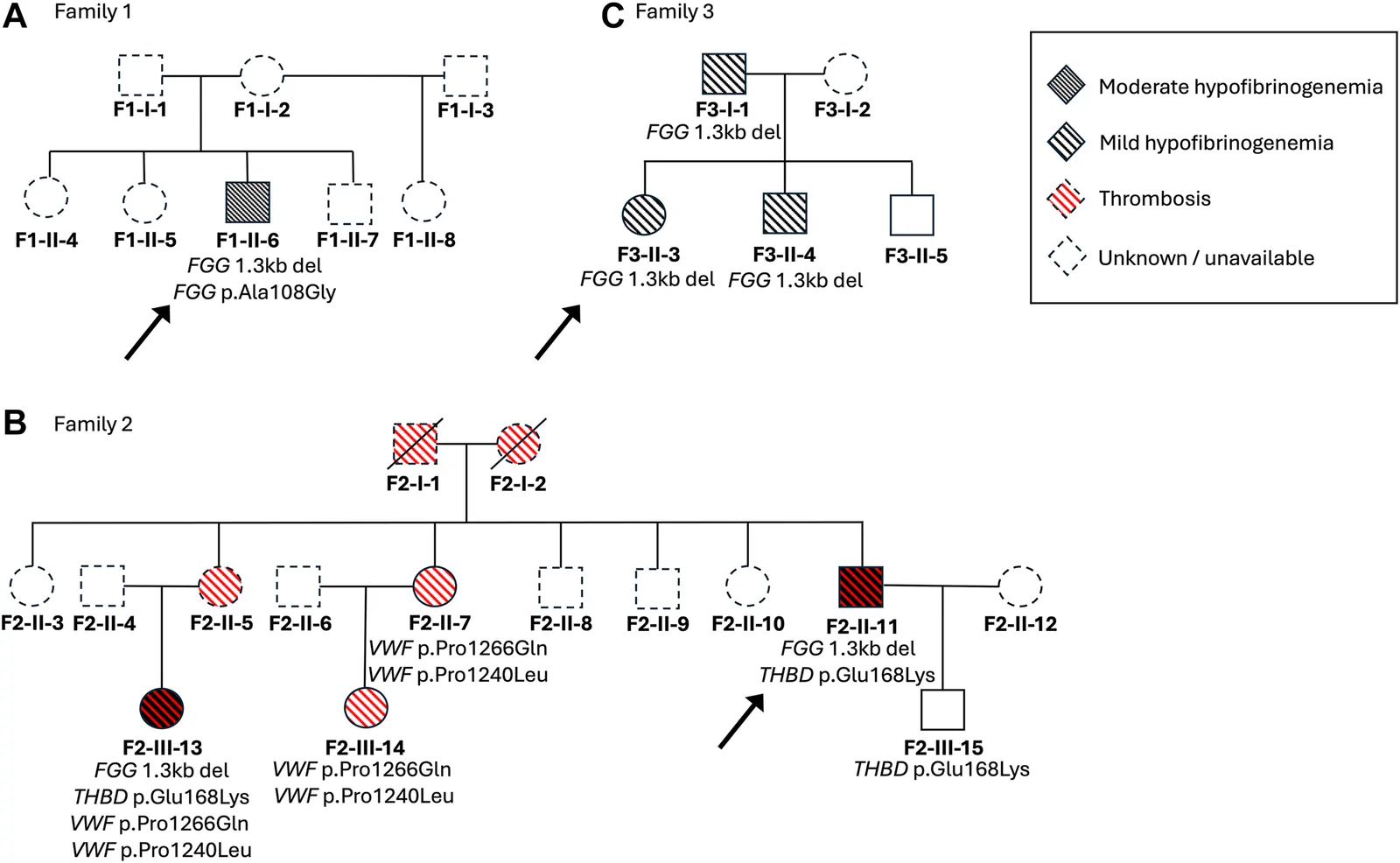
Family pedigrees of the probands. (A) Family 1, (B) family 2, and (C) family 3. Squares indicate males and circles indicate females. Black shading indicates individuals with mild or moderate hypofibrinogenemia. Red shading indicates a personal history of thrombosis. Individuals with both features are indicated by combined shading. Slashed symbols indicate deceased individuals. Dashed symbols indicate individuals for whom samples were unavailable or clinical data were missing. The *FGG* deletion, *FGG* p.Ala108Gly, and *THBD* p.Glu168Lys are indicated below the relevant individuals. Arrows indicate the probands.

**Table:** Clinical characteristics and genetic findings in the 3 families.

Family	Individual	Fibrinogen Antigen (g/L)	Fibrinogen (Clauss) (g/L)	Primary fibrinogen genotype^a^	Additional variants of interest^b^	Bleeding	Thrombosis
1	**F1-II-6**	**0.7**	**0.6**	**Heterozygous *FGG* 1.3 kb del** **Heterozygous *FGG* c.323C>G (p.Ala108Gly)**	**None**	**Mild (oral cavity, epistaxis)**	**None**
2	F2-II-7	3.7	3.6	WT	Heterozygous *VWF* c.3797C>A (p.Pro1266Gln) Heterozygous *VWF* c.3719C>T (p.Pro1240Leu)	None	3 × DVT; 2 × PE
	**F2-II-11**	**1.6**	**1.5**	**Heterozygous *FGG* 1.3 kb del**	**Heterozygous *THBD* c.502G>A (p.Glu168Lys)**	**None**	**3** × **DVT; 1** × **AT**
	F2-III-13	NA	1.5	Heterozygous *FGG* 1.3 kb del	Heterozygous *THBD* c.502G>A (p.Glu168Lys) Heterozygous *VWF* c.3797C>A (p.Pro1266Gln) Heterozygous *VWF* c.3719C>T (p.Pro1240Leu)	None	3 × DVT
	F2-III-14	3.4	3.0	WT	Heterozygous *VWF* c.3797C>A (p.Pro1266Gln) Heterozygous *VWF* c.3719C>T (p.Pro1240Leu)	None	2 × DVT
	F2-III-15	2.5	2.5	WT	Heterozygous *THBD* c.502G>A (p.Glu168Lys)	None	None
3	F3-I-1	1.9	1.6	Heterozygous *FGG* 1.3 kb del	None	Mild (cutaneous, oral cavity; postextraction); muscle hematoma	None
	**F3-II-3**	**1.2**	**1.1**	**Heterozygous *FGG* 1.3 kb del**	**None**	**Mild (cutaneous, oral cavity)**	**None**
	F3-II-4	1.2	1.2	Heterozygous *FGG* 1.3 kb del	None	Muscle hematoma	None

Individuals in bold represent probands in this study.

AT, arterial thrombosis; DVT, deep vein thrombosis; NA, not available; PE, pulmonary embolism; WT, wild type.

a *FGG* transcript NM_021870.3; the FGG 1.3 kb deletion corresponds to GRCh38 NC_000004.12:g.154612005_154613347del.

b *THBD* transcript NM_000361.3; *VWF* transcript NM_000552.5.

### 3.1 Clinical presentation

All ages are reported at the time of clinical evaluation. Self-reported race, ethnicity, and other sociocultural variables were unavailable for most participants, limiting demographic characterization of the study population.

#### 3.1.1 Family 1

The proband, F1-II-6 (21 years old), was identified during preoperative screening with reduced fibrinogen levels (antigenic 0.7 g/L and functional 0.6 g/L), consistent with moderate hypofibrinogenemia [4]. Clinical history revealed a mild bleeding phenotype, characterized by mucocutaneous bleeding, including oral cavity bleeding and epistaxis. The proband had previously undergone surgical treatment for phimosis and sustained a left arm fracture, both without replacement therapy or reported bleeding complications. The proband’s mother (F1-I-2) and sister (F1-II-5) were reported to have normal fibrinogen levels; no additional family members were available for clinical or genetic evaluation.

#### 3.1.2 Family 2

The proband, F2-II-11 (54 years old), had no history of bleeding but presented with recurrent idiopathic thrombotic events, including deep vein thrombosis (DVT) in 2011, 2015, and 2020, treated with vitamin K antagonists or rivaroxaban, and arterial thrombosis in 2021, treated with aspirin and atorvastatin. Subsequent laboratory investigation identified reduced fibrinogen levels (antigenic 1.6 g/L and functional 1.5 g/L), consistent with mild hypofibrinogenemia. A strong family history of thrombosis was present. Among relatives available for familial screening, the proband’s sister, F2-II-7 (70 years old), had postoperative and spontaneous episodes of DVT as well as postoperative pulmonary embolism, with normal fibrinogen levels; she had additional cardiovascular risk factors, including hypertension and obesity (body mass index, >30 kg/m^2^). Two nieces also had a history of thrombosis. F2-III-13 (54 years old) had mild hypofibrinogenemia and recurrent DVT, whereas F2-III-14 (43 years old) had normal fibrinogen levels and 2 episodes of DVT. No additional cardiovascular risk factors were reported in either individual. In contrast, the proband’s son, F2-III-15 (18 years old), had no history of thrombosis and normal fibrinogen levels.

#### 3.1.3 Family 3

The proband, F3-II-3 (30 years old), was identified through pregnancy screening and had mild hypofibrinogenemia (antigenic 1.2 g/L and functional 1.1 g/L). She reported a mild bleeding phenotype, characterized by mucocutaneous bleeding including oral bleeding, but experienced a successful pregnancy without the need for supplementation. Two additional family members were available for testing: her father (F3-I-1, 51 years old) and brother (F3-II-4, 27 years old), both of whom had mild hypofibrinogenemia. Her father reported postsurgical oral bleeding and post-traumatic muscle hematomas, whereas her brother reported post-traumatic muscle hematomas.

### 3.2 Genotyping

#### 3.2.1 Proband analysis

The 3 apparently unrelated probands were initially investigated using WES to assess variation in the fibrinogen genes, as well as other coagulation and fibrinolytic genes, followed by targeted ONT long-read sequencing to resolve a suspected structural variant (Figure 1).

#### 3.2.2 Identification and resolution of a 1.3 kb *FGG* deletion

Read-depth analysis of WES data revealed a consistent reduction across exons 1-3 of *FGG* in all 3 probands (F1-II-6, F2-II-11, and F3-II-3), suggesting a putative CNV of approximately 0.6 kb (chr4:155,533,169-155,533,761, GRCh37) (Figure 2). Because WES could not define the full extent or breakpoints of the suspected rearrangement, or definitively exclude a false-positive read-depth signal, targeted ONT long-read sequencing was subsequently performed.

**Figure 2:**
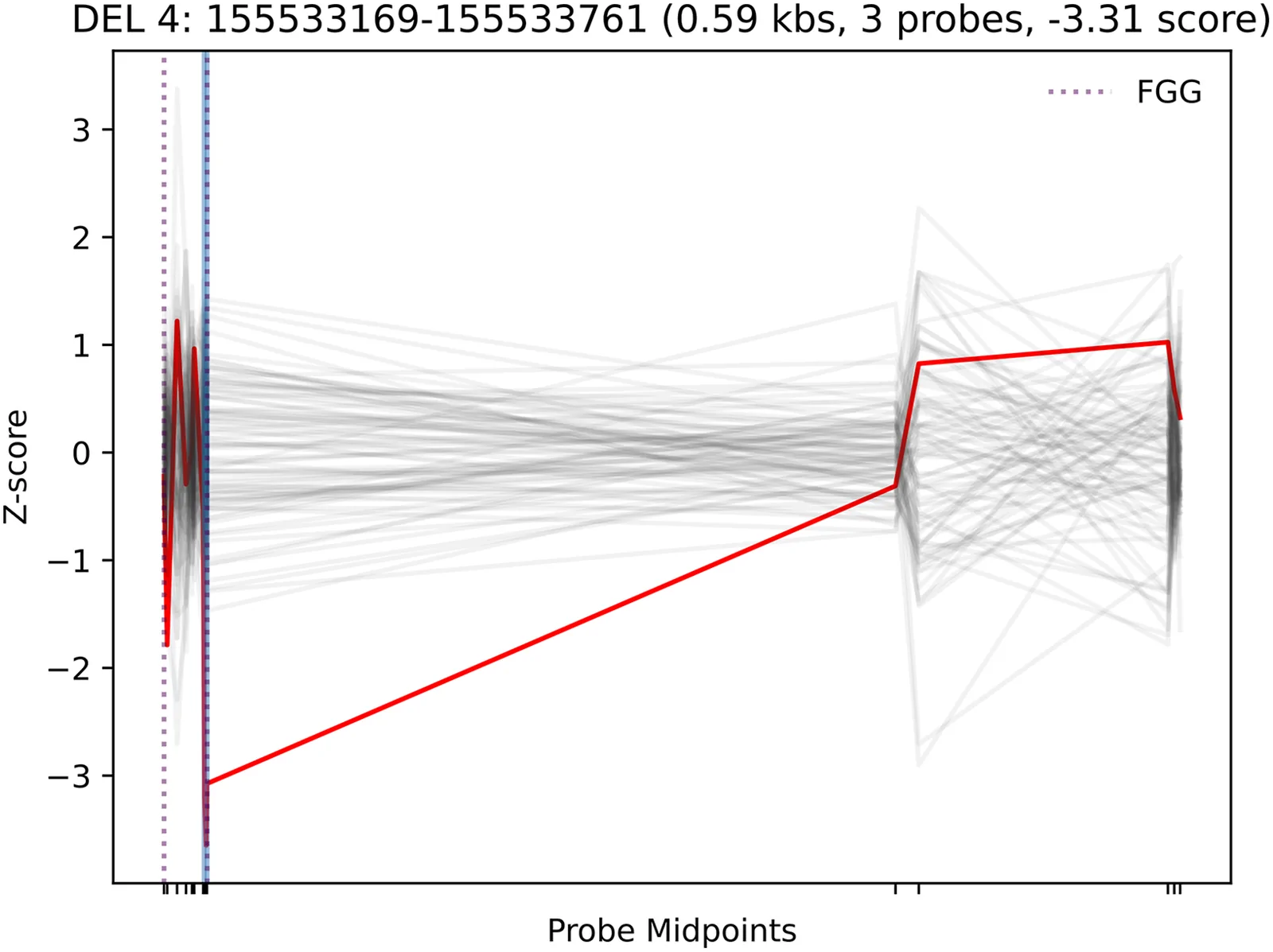
Detection of a potential deletion in *FGG* from whole-exome sequencing data. Copy number variation analysis was performed using an in-house read depth–based algorithm combining CoNIFER and XHMM. CoNIFER calculates normalized read-depth *z* scores for each exon across samples, which are then analyzed by XHMM using a hidden Markov model to identify stretches of deleted or duplicated exons. The plot shows *z* scores (*y*-axis) across probe midpoints (*x*-axis) for the region encompassing *FGG*. The red line represents the sample of interest, showing a marked decrease in normalized read depth across 3 consecutive probes corresponding to a potential 0.59 kb deletion (DEL 4:155533169-155533761), which was later shown to be a 1.3 kb deletion extending into the upstream genomic region and therefore not fully captured by WES. Gray lines indicate reference samples.

Targeted long-read sequencing confirmed a heterozygous CNV in the 3 probands and resolved it as a 1343 bp deletion (NC_000004.12:g.154612005_154613347del, GRCh38). The deletion spans chr4:154,612,005-154,613,347 (GRCh38), encompassing the defined *FGG* promoter region and extending into intron 3 downstream of exon 3 (Figure 3). As this deletion removes the promoter and 5′ coding region of *FGG*, it is predicted to abolish expression from the affected allele. Under the ACMG/AMP framework for variant interpretation, the pathogenic very strong 1 (PVS1) criterion was therefore applied, as loss of function is an established mechanism of quantitative fibrinogen deficiency. Breakpoint analysis showed that both junctions lie within low-complexity A/T-rich sequences, including mononucleotide runs and short tandem repeats. No structural variant with matching breakpoints or comparable genomic extent was identified in the queried databases, supporting the novelty of the deletion.

**Figure 3:**
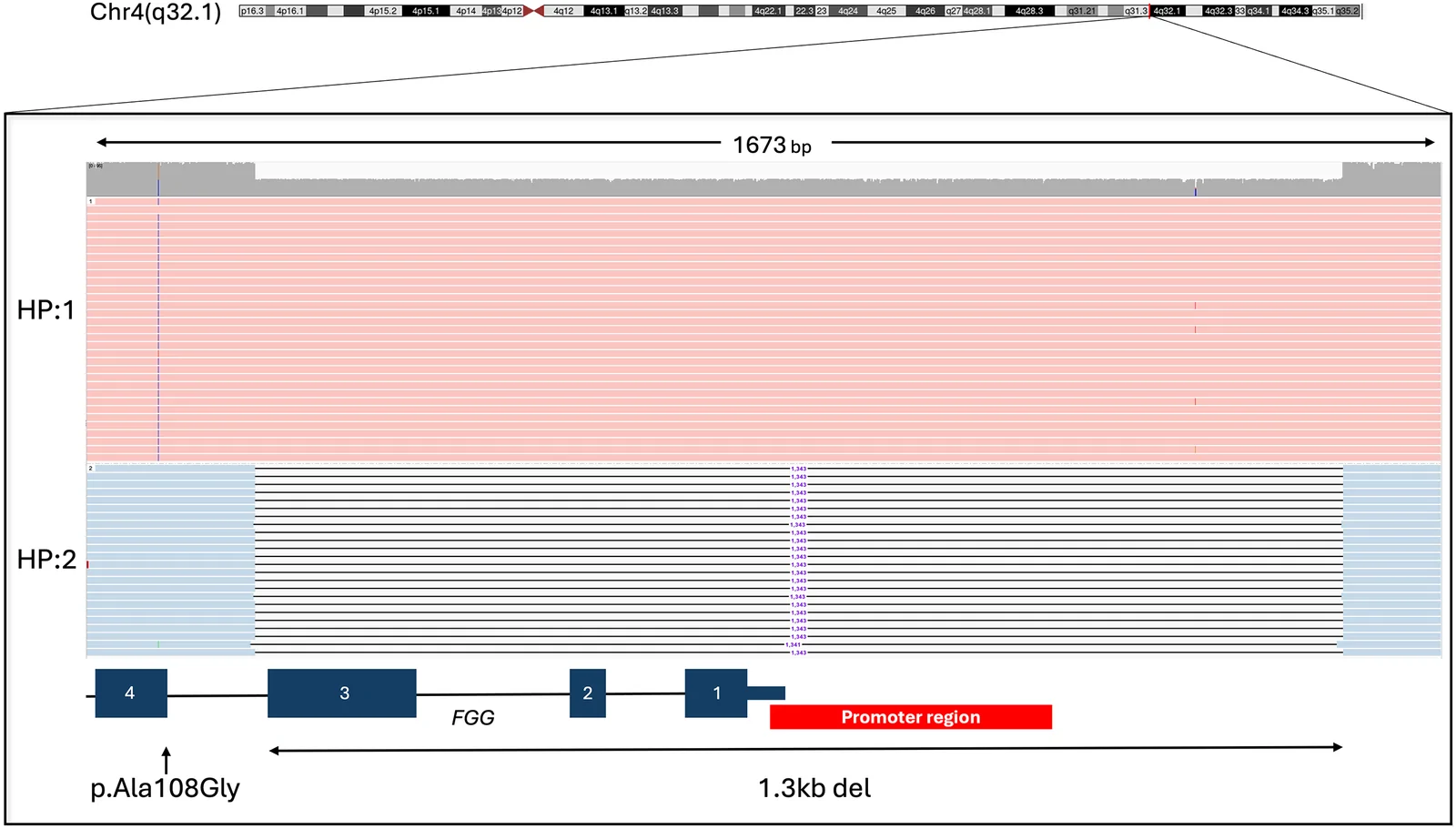
IGV analysis of haplotagged Oxford Nanopore long reads confirming and resolving the *FGG* deletion. Long-read sequencing data from individual F1-II-6 are shown, with reads colored by haplotype (HP; pink = HP:1, blue = HP:2). HP:2 carries the 1.3 kb deletion, visible as a reduction in coverage (gray) and absence of aligned sequence (gaps in blue reads). Long reads enable precise resolution of the deletion breakpoints, confirming that the deletion spans the defined *FGG* promoter region and exons 1-3 (shown below as red and blue boxes). In contrast, HP:1 harbors the single-nucleotide variant FGG c.323C>G p.Ala108Gly (vertical blue line within pink reads), confirming compound heterozygosity in this individual. The same 1.3 kb deletion was also identified in additional individuals (data not shown).

ONT sequencing enabled phasing across an approximately 18 kb region of the *FGG* locus and identified a deletion-bearing haplotype comprising the 1.3 kb *FGG* deletion and 13 additional variants shared by all 3 probands. In contrast, the non–deletion-bearing haplotypes showed distinct variant patterns between individuals (Figure 4, Supplementary Table). Together, these findings indicate that the deletion is present on an identical haplotype block in all 3 individuals, whereas the alternate haplotypes are distinct.

**Figure 4:**
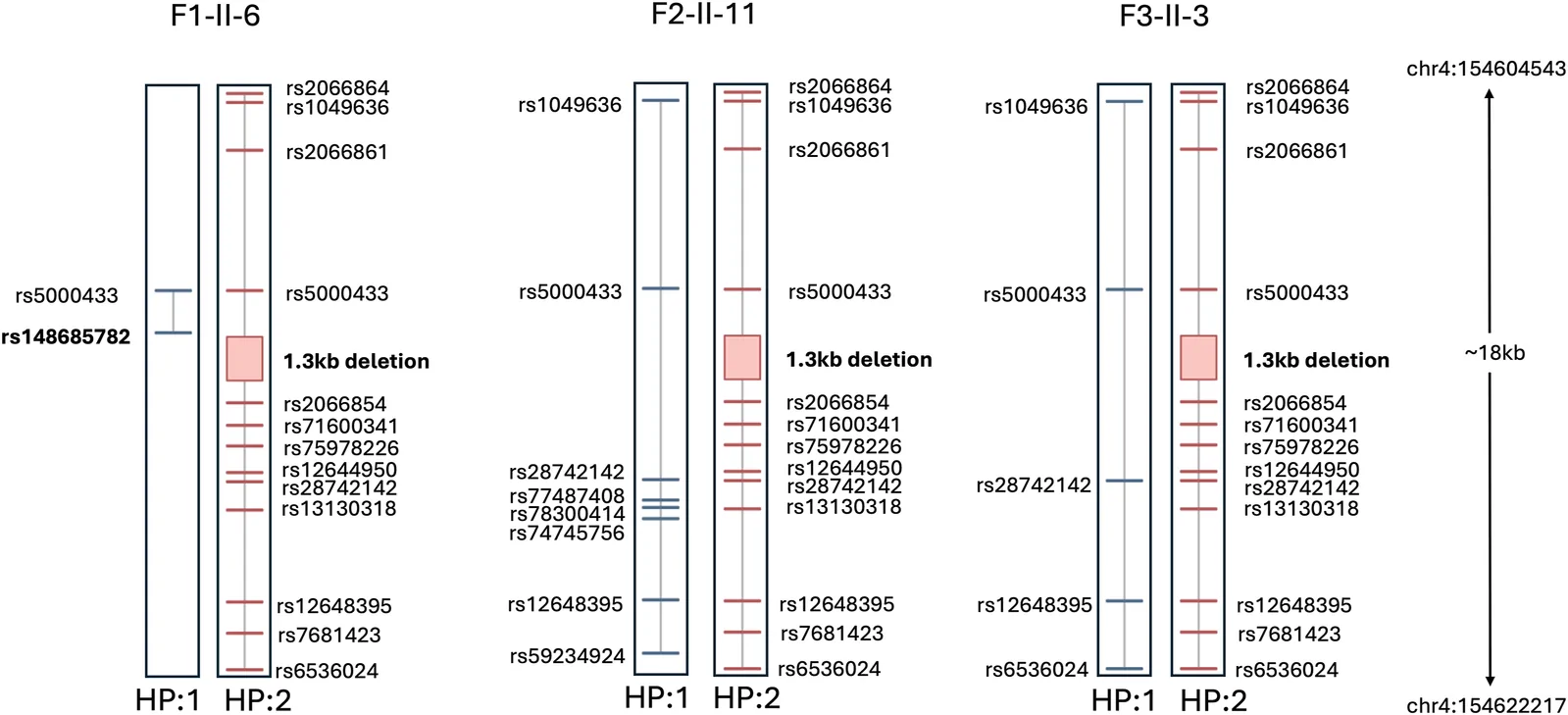
Haplotype structure across the *FGG* locus in probands. Single-nucleotide variants and deletions identified within an 18 kb region spanning *FGG* are shown for individuals F1-II-6, F2-II-11, and F3-II-3. Variants were identified and phased using Oxford Nanopore long-read sequencing across *FGG*. Variants present on HP:2 are shared across all 3 individuals, consistent with inheritance of a common haplotype carrying a shared 1.3 kb deletion in *FGG*. In contrast, variants on the alternate chromosome (HP:1 in each individual) show heterogeneous patterns between individuals. Colors indicate phased haplotypes (HP:1, blue; HP:2, red). Variants highlighted in bold represent variants of interest in this study. Genomic visualizations were generated in R (v4.3.1; RStudio) using the Gviz package (Bioconductor).

#### 3.2.3 Additional variants of interest

Two additional single-nucleotide variants of interest were identified by WES in 2 of the 3 probands. In F1-II-6, the variant *FGG* NM_021870.3:c.323C>G (p.Ala108Gly) (rs148685782) was detected. Phasing of targeted ONT long reads demonstrated that this missense variant and the 1.3-kb *FGG* deletion were located on opposite haplotypes, establishing compound heterozygosity in F1-II-6 (Figure 3). *FGG* p.Ala108Gly is rare in gnomAD v4.1 (allele frequency, 3.0 × 10^–3^) but has been reproducibly associated with reduced plasma fibrinogen levels in population-based studies, with an estimated effect of approximately 0.8-1 standard deviations per allele [24], [25]. It has also been reported in individuals with hypofibrinogenemia, both in heterozygous and compound heterozygous states [8], [26], [27], [28]. A previous report described p.Ala108Gly in trans with a 14.8 Mb deletion encompassing the entire fibrinogen gene cluster in an individual with similarly reduced but detectable fibrinogen levels [15]. Given its apparent hypomorphic effect and the absence of direct functional evidence, no formal ACMG/AMP classification was assigned. Its relevance was instead interpreted in light of the published evidence and the confirmed compound heterozygous configuration in F1-II-6.

In F2-II-11, WES identified a rare variant of unknown significance in *THBD*, NM_000361.3:c.502G>A (p.Glu168Lys) (rs922265393). This variant is extremely rare in gnomAD v4.1.0 (allele frequency, 3.2 × 10^–6^) and has not, to our knowledge, previously been reported in association with human disease. In silico predictions are discordant, with a high CADD score (28.3) and AlphaMissense classification of likely pathogenic (0.744) but a low REVEL score (0.181). Rare *THBD* variants have been associated with bleeding and venous thromboembolism, although variant-level evidence for p.Glu168Lys is lacking [29], [30], [31]. Given the absence of variant-specific functional evidence and limited segregation data, its clinical significance remains uncertain. Its potential structural effect was therefore explored by in silico modeling, as described below.

No other variants of immediate relevance were identified in proband F3-II-3. No common inherited thrombophilia variants, including *F5* Leiden (rs6025) and *F2* G20210A (rs1799963), were detected in the probands.

#### 3.2.4 Additional family analysis

Where available, additional family members were investigated using a combination of WES, ONT sequencing, and/or Sanger sequencing to assess segregation of the identified variants (Table). No additional family members were available for genetic testing in family 1.

In family 2, the proband’s sister, F2-II-7, did not carry the *FGG* deletion but carried 2 heterozygous *VWF* variants: NM_000552.5:c.3797C>A (p.Pro1266Gln) (rs61749370) and NM_000552.5:c.3719C>T (p.Pro1240Leu) (rs150576611). The proband’s niece, F2-III-13, was heterozygous for the 1.3 kb *FGG* deletion and also carried the *THBD* p.Glu168Lys variant identified in the proband, together with the same 2 *VWF* variants. The other niece, F2-III-14, did not carry the *FGG* deletion but carried both *VWF* variants. The proband’s son, F2-III-15, did not carry the *FGG* deletion but was heterozygous for *THBD* p.Glu168Lys. All additional individuals tested were negative for the common inherited thrombophilia variants *F5* Leiden and *F2* G20210A.

The 2 *VWF* variants identified in F2-II-7, F2-III-13, and F2-III-14, but not in the proband, are reported for completeness. *VWF* p.Pro1266Gln has been associated with the Malmö/New York subtype of type 2B von Willebrand disease, although it is currently classified as a variant of uncertain significance by the ClinGen von Willebrand Disease Expert Panel [32], [33]. *VWF* p.Pro1240Leu has been reported only once in compound heterozygosity and without supporting functional evidence [34]. Given the absence of VWF antigen/activity data, the lack of thrombocytopenia (data not shown), and the absence of an established association with thrombosis, neither variant was considered a likely contributor to the thrombotic phenotype in this family.

In family 3, WES and targeted ONT long-read sequencing confirmed that both the proband’s father, F3-I-1, and brother, F3-II-4, were heterozygous carriers of the *FGG* deletion. No additional variants of clinical relevance were identified.

### 3.3 Kinship analysis

Given that all 3 probands carried the same deletion, kinship analysis was performed using KING to assess whether they were related. Pairwise kinship coefficients were below 0.0442, indicating that no third-degree or closer relationship was detected between the probands.

### 3.4 In silico assessment of *THBD* p.Glu168Lys

To investigate the possible relevance of the rare *THBD* p.Glu168Lys variant in the context of the thrombotic phenotype observed in family 2, exploratory structural modeling was performed using AlphaFold3. The p.Glu168Lys substitution lies near the boundary of the C-type lectin-like domain (amino acids 31-169), a region susceptible to proteolytic cleavage [35]. A nearby variant, p.Cys175Ser, has been reported in an individual with unprovoked venous thrombosis who also carried the prothrombin *F2* G20210A risk variant. *In vitro* studies showed that p.Cys175Ser modestly reduced protein C and thrombin-activatable fibrinolysis inhibitor (TAFI) activation, likely through altered thrombomodulin structure [29]. Exploratory in silico visualization revealed a local alteration in surface contour resulting from the change in side-chain properties (Supplementary Figure). However, this analysis does not establish that the substitution meaningfully alters thrombomodulin folding or function. In the absence of functional validation and further segregation data, the clinical significance of p.Glu168Lys remains uncertain, and no firm conclusions can be drawn regarding its effect on thrombomodulin function or thrombotic risk.

## 4 Discussion

This study identifies and characterizes a novel heterozygous 1.3 kb deletion affecting *FGG* in 3 apparently unrelated families with hypofibrinogenemia. Although WES suggested a CNV through reduced read depth across *FGG* exons 1 to 3, targeted ONT long-read sequencing was required to validate the rearrangement, define its breakpoints, establish its heterozygous state, and phase it with nearby variants. We show that the deletion encompasses the *FGG* promoter and 5′ coding region and is therefore predicted to abolish expression from the affected allele.

Although the deletion was identified in 3 families from the same geographical region, kinship analysis detected no third-degree or closer relationship between the probands. However, haplotype analysis using ONT long reads showed that all 3 probands carried the deletion on an identical 18 kb haplotype block. This supports inheritance from a more distant common founder and suggests that the deletion represents a nonrecurrent rearrangement rather than independent events. In this respect, the deletion differs from the previously described large *FGA* deletion, which occurs on multiple distinct haplotypes and is therefore likely recurrent [10].

Structural variants are not randomly distributed across the genome, and breakpoint junctions often localize to regions of intrinsic genomic instability. Here, precise breakpoint mapping showed that this deletion occurs within A/T-rich low-complexity sequences, including mononucleotide runs and short tandem repeats, consistent with sequence contexts known to promote replication stress and polymerase slippage, thereby predisposing to genomic instability [36]. Its absence from public databases supports the apparent novelty of this event, although rare structural variants remain under-represented in resources that are largely based on short-read sequencing data.

Despite carrying the same primary *FGG* deletion, affected individuals showed variable fibrinogen levels and clinical phenotypes, with recurrent thrombosis observed in family 2, underscoring the well-recognized heterogeneity of CFDs. In F1-II-6, haplotype phasing of targeted ONT long reads demonstrated that the *FGG* deletion and p.Ala108Gly were located on opposite haplotypes, establishing compound heterozygosity despite the absence of samples from additional family members for segregation analysis. This compound heterozygous configuration provides a plausible explanation for the more marked reduction in fibrinogen levels to below 1 g/L. In contrast, heterozygous carriers of the deletion with a wild-type *FGG* allele had only mild hypofibrinogenemia, with fibrinogen levels above 1 g/L. This interpretation is consistent with population-based studies showing that p.Ala108Gly is associated with reduced plasma fibrinogen levels as well as a prior report of the same variant in trans with a large fibrinogen gene deletion in an individual with markedly reduced, but detectable, fibrinogen levels [15], [24], [25]. Together, these observations support a hypomorphic effect of p.Ala108Gly, whereby the variant reduces but does not abolish fibrinogen production and, when present in trans with a null allele, results in hypofibrinogenemia rather than afibrinogenemia. The fibrinogen level below 1 g/L in F1-II-6 may therefore confer a greater bleeding risk than that observed in heterozygous deletion carriers with a wild-type *FGG* allele and higher fibrinogen levels [37].

The recurrent thrombotic events observed in family 2 were of particular interest but remain difficult to interpret in light of the available genetic findings. F2-II-11, who had mild hypofibrinogenemia, experienced multiple episodes of DVT and 1 arterial thrombosis. Although thrombosis is recognized in quantitative fibrinogen disorders, affecting approximately 10% to 20% of individuals with hypofibrinogenemia, the underlying mechanisms remain incompletely understood, and there is no clear genotype-phenotype correlation or consistent relationship with fibrinogen levels [6]. In keeping with this, although impaired thrombin sequestration due to reduced circulating fibrinogen may contribute to a prothrombotic tendency, this individual did not have the lowest fibrinogen levels in the study and was the only proband to experience thrombosis. This prevents definitive attribution of the thrombotic phenotype to fibrinogen deficiency alone and suggests that additional genetic or environmental factors could be involved, particularly given the strong family history of thrombosis.

The rare *THBD* p.Glu168Lys variant remains a candidate modifier of thrombotic risk because of the role of thrombomodulin in anticoagulation and fibrinolysis, although variant-specific evidence remains limited. Furthermore, segregation with thrombosis was incomplete: it was present in F2-II-11 and F2-III-13, both of whom also carried the *FGG* deletion and had experienced thrombosis, but was absent from F2-II-7 and F2-III-14, who had thrombosis without either variant. It was also identified in F2-III-15, an unaffected 18-year-old who did not carry the deletion, although his young age limits interpretation of the absence of thrombosis at present. A possible explanation is that the combination of the *FGG* deletion and *THBD* p.Glu168Lys contributes to thrombotic risk in F2-II-11 and F2-III-13 by altering hemostatic balance. However, this remains speculative, is not currently supported by functional evidence, and does not explain thrombosis in relatives lacking both variants. Additional genetic, acquired, or environmental factors are therefore likely to contribute. Further interpretation will require functional comparison of wild-type and p.Glu168Lys thrombomodulin, including assessment of thrombomodulin expression, thrombin-dependent activation of protein C and TAFI, and inhibition of thrombin generation, as previously applied to other *THBD* variants [29]. These studies should be accompanied by broader segregation analysis and evaluation in larger patient cohorts. The *VWF* variants identified in family 2 were reported for completeness and were not considered likely contributors to the thrombotic phenotype.

In family 3, F3-II-3, like her father and brother, carried only the *FGG* deletion and had mildly reduced fibrinogen levels. All 3 reported mild bleeding symptoms, but none had experienced major bleeding or thrombotic complications, and no additional variants of immediate clinical relevance were identified. This supports previous observations that retention of a wild-type allele and fibrinogen levels above approximately 1 g/L are generally sufficient to protect against major bleeding episodes [37].

Several limitations should be acknowledged. This study included only 3 families, reflecting both the rarity of CFDs and the apparent rarity of this *FGG* deletion. In addition, not all family members were available for testing, resulting in incomplete segregation data. Functional assays were not available to assess the effect of *THBD* p.Glu168Lys on thrombomodulin expression or function, limiting interpretation of its possible contribution to thrombotic risk. Environmental and acquired risk factors for thrombosis could not be comprehensively assessed for all individuals, although these may contribute to inter- and intrafamilial variability in clinical phenotypes. These limitations highlight the difficulty of explaining the observed clinical heterogeneity using genetic data alone. Finally, although WES enabled assessment of coding variants in hemostasis-related genes, it may not capture additional contributors outside the analyzed regions, including noncoding or regulatory variants, complex structural variation beyond the targeted locus, or broader polygenic risk. Genome-wide long-read sequencing may facilitate the detection of additional structural variants, including variants affecting regulatory elements that are not captured by WES. Future studies should include more extensive segregation analysis, functional assessment of candidate variants, and evaluation of genetic and environmental modifiers in larger cohorts of patients with hypofibrinogenemia and thrombotic phenotypes.

In summary, WES and targeted long-read sequencing provided complementary approaches for investigating CFDs. WES revealed a copy-number signal and enabled assessment of additional coding variants, whereas targeted ONT sequencing resolved the 1343 bp *FGG* deletion, defined its breakpoints, and phased it with nearby variants. These analyses clarified the predicted loss-of-function consequence of the deletion, supported inheritance from a common founder, and demonstrated that *FGG* p.Ala108Gly was present in trans with the deletion in F1-II-6 without requiring samples from additional family members. This study therefore illustrates the value of targeted ONT long-read sequencing for resolving WES-derived copy-number signals and fully characterizing structural variants.

## Figures and Tables

**Supplementary Figure 1:**
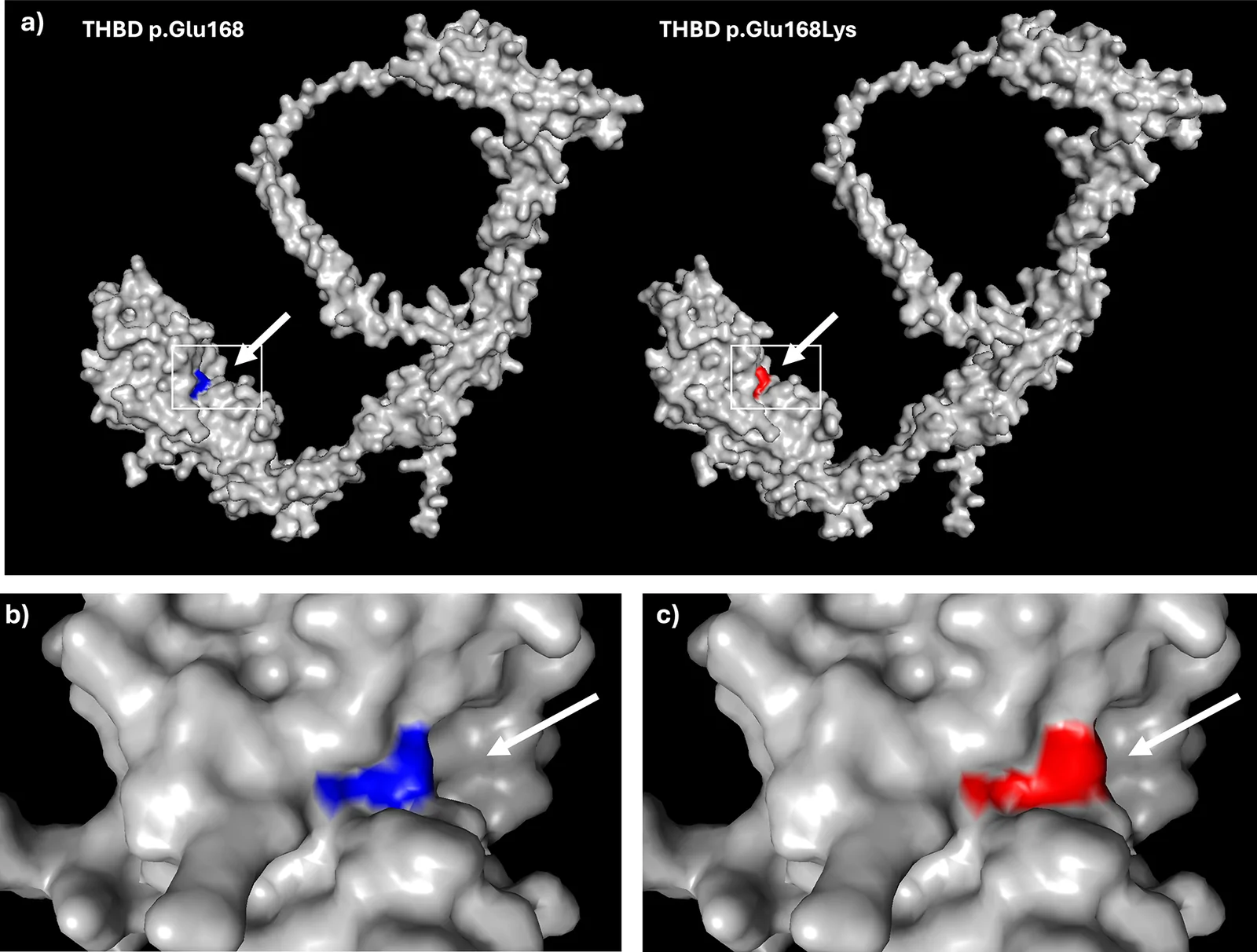
Surface representations of the AlphaFold3-predicted thrombomodulin structure before and after in silico introduction of p.Glu168Lys. (a) Full-length wild-type protein (left) and p.Glu168Lys variant (right). The location of residue 168 is indicated by a white box and arrow. (b) Magnified view of the region surrounding Glu168 in the wild-type protein (blue). (c) Corresponding magnified view of the p.Glu168Lys variant (red). Visualisation shows a local difference in surface contour reflecting the altered side-chain properties.
