# Supplementary material for: Targeted long-read sequencing identifies a novel 1.3 kb FGG deletion associated with variable bleeding and thrombotic phenotypes in hypofibrinogenemia
Source: Res Pract Thromb Haemost. 2026 Oct 1;10(7):107001. doi: 10.1016/j.rpth.2026.107001 (PMC13734769; doi:10.1016/j.rpth.2026.107001)
Supplement: Supplementary file 1 [file mmc1.docx]

Supplementary material

**Supplementary Figure 1. Surface representations of the AlphaFold3-predicted thrombomodulin structure before and after in silico introduction of p.Glu168Lys.**

*(a) Full-length wild-type protein (left) and p.Glu168Lys variant (right). The location of residue 168 is indicated by a white box and arrow. (b) Magnified view of the region surrounding Glu168 in the wild-type protein (blue). (c) Corresponding magnified view of the p.Glu168Lys variant (red). Visualisation shows a local difference in surface contour reflecting the altered side-chain properties.*

**Supplementary Table 1. Phased haplotype structure across the *FGG* locus.**

| **Variant^*^** | **Variant type** | **rsID** | **AF** ^†^ | **F1-II-6 (HP1\|HP2)** ^‡^ | **F2-II-11 (HP1\|HP2)** ^‡^ | **F3-II-3 (HP1\|HP2)** ^‡^ |
| --- | --- | --- | --- | --- | --- | --- |
| chr4:154604543:G>A | SNV | rs2066864 | 0.238 | 0\|1 | 0\|1 | 0\|1 |
| chr4:154604818:G>A | SNV | rs1049636 | 0.6894 | 0\|1 | 1\|1 | 1\|1 |
| chr4:154606284:C>T | SNV | rs2066861 | 0.2627 | 0\|1 | 0\|1 | 0\|1 |
| chr4:154610588:G>A | SNV | rs5000433 | 0.9985 | 1\|1 | 1\|1 | 1\|1 |
| **chr4:154611883:G>C** | **SNV** | **rs148685782** | 0.003031 | **1\|0** | **0\|0** | **0\|0** |
| **chr4:154612005-154613347:del** | **DEL (1.3 kb)** | **-** | **-** | **0\|1** | **0\|1** | **0\|1** |
| chr4:154614029:T>A | SNV | rs2066854 | 0.2564 | 0\|1 | 0\|1 | 0\|1 |
| chr4:154614720:ACT>A | DEL (2 bp) | rs71600341 | 0.2548 | 0\|1 | 0\|1 | 0\|1 |
| chr4:154615353:GA>A | DEL (1 bp) | rs75978226 | 0.2574 | 0\|1 | 0\|1 | 0\|1 |
| chr4:154616169:G>A | SNV | rs12644950 | 0.2566 | 0\|1 | 0\|1 | 0\|1 |
| chr4:154616449:C>T | SNV | rs28742142 | 0.6968 | 0\|1 | 1\|1 | 1\|1 |
| chr4:154617078:C>T | SNV | rs77487408 | 0.1401 | 0\|0 | 1\|0 | 0\|0 |
| chr4:154617304:A>G | SNV | rs78300414 | 0.1314 | 0\|0 | 1\|0 | 0\|0 |
| chr4:154617318:T>G | SNV | rs13130318 | 0.232 | 0\|1 | 0\|1 | 0\|1 |
| chr4:154617648:C>T | SNV | rs74745756 | 0.136 | 0\|0 | 1\|0 | 0\|0 |
| chr4:154620137:C>T | SNV | rs12648395 | 0.712 | 0\|1 | 1\|1 | 1\|1 |
| chr4:154621096:C>T | SNV | rs7681423 | 0.2616 | 0\|1 | 0\|1 | 0\|1 |
| chr4:154621774:G>A | SNV | rs59234924 | 0.1832 | 0\|0 | 1\|0 | 0\|0 |
| chr4:154622217:T>C | SNV | rs6536024 | 0.4654 | 0\|1 | 0\|1 | 1\|1 |

** Single-nucleotide variants (SNVs) and deletions (DEL) within an approximately 18 kb region (chr4:154.60–154.62 Mb) spanning FGG in F1-II-6, F2-II-11, and F3-II-3 were identified using long-read nanopore sequencing and phased with WhatsHap.* † *Population allele frequencies (AF) are from gnomAD v4.1.0 (global).* ‡ *Genotypes are displayed as phased alleles (HP1|HP2), where 0 indicates the reference allele and 1 indicates the alternate allele. Variants present on HP2 (0|1) are shared across all three individuals, consistent with inheritance of an identical haplotype block, whereas variants on the other chromosome in each individual (HP1; 1|0) show heterogeneous sharing patterns between individuals. Homozygous variants (1|1) are present on both chromosomes. The region includes a shared 1.3 kb deletion, represented by its genomic interval, which is present on HP2 in all three individuals. Variants in bold represent variants of interest in this study.*
